# No clear benefit or drawback to the use of closed drainage after primary total knee arthroplasty: a systematic review and meta-analysis

**DOI:** 10.1186/s12891-016-1039-2

**Published:** 2016-04-26

**Authors:** Hai-bo Si, Ti-min Yang, Yi Zeng, Bin Shen

**Affiliations:** Department of Orthopaedics, West China Hospital, Sichuan University, 37# Guoxue Road, Chengdu, Sichuan Province 610041 China

**Keywords:** Total knee arthroplasty, Drainage, Systematic review, Meta-analysis

## Abstract

**Background:**

Closed drainage after primary total knee arthroplasty (TKA) has been used routinely for many decades, but controversies have arisen in recent years. The purposes of this study were to compare the clinical outcomes of closed drainage with nondrainage after primary TKA; and to assess the benefit and drawback of closed drainage.

**Methods:**

Electronic databases (PubMed/Medline, CENTRAL, Embase and Web of Science) were systematically searched for randomised controlled trials (RCTs) that investigated the efficacy and risks of closed drainage after primary TKA. Two investigators independently reviewed studies for eligibility, assessed the risk of bias and extracted the data. A meta-analysis was then performed using Review Manager Software.

**Results:**

Twelve RCTs totalling 889 TKAs were identified. No significant differences in infection rate or blood loss were found between the closed drainage and nondrainage TKAs, and there was also no significant difference in haematoma formation, deep venous thrombosis, postoperative VAS score or range of motion between the two groups.

**Conclusions:**

There appears to be no clear benefit or drawback to the use of closed drainage after primary TKA. Improving the use of closed drainage might provide better outcomes.

## Background

In 1961, Waugh and Stinchfield were the first to advocate the use of drainage after orthopaedic surgery [1]. In particular, they observed that there was less pain, swelling and infection in patients whose wounds were drained, as well as better healing of the soft tissues and quicker mobilisation of the extremities. Since then, closed drainage has been used routinely for many years to prevent haematoma formation [2], reduce the risk of infection and accelerate wound healing [3, 4].

In the past decades, many studies have compared closed drainage with nondrainage after primary total knee arthroplasty (TKA). Conflicting results have been reported, and an increasing number of studies have demonstrated no benefit to the use of closed drainage [5–8]. Those who use closed drainage argue that it limits haematoma formation, decreases the risk of infection and improves range of motion (ROM) after surgery, limitations that can possibly necessitate additional surgery [6]. In contrast, those who do not use drainage argue that it can serve as a portal for bacteria and increase the infection rate [4, 9], postoperative blood loss, need for blood transfusion [10, 11], and total costs [12, 13]. Whether to use closed drainage after primary TKA remains controversial; thus, an evidence-based study evaluating the outcomes of closed drainage might be helpful for joint surgeons.

The objectives of this study, therefore, were to compare the clinical outcomes, of which the infection rate and amount of blood loss were the most important, between closed drainage and nondrainage patients undergoing primary TKA and to assess the benefits and disadvantages of closed drainage.

## Methods

### Search strategy and selection criteria

We followed the recommendations of the Cochrane Collaboration in executing our search strategy [14]. The electronic databases of PubMed/Medline (1966 to February 2016), CENTRAL (The Cochrane Central Register of Controlled Trials, Issue 2 of 12, February 2016), Embase (1984 to February 2016, Exclude Medline Journals) and Web of Science (1994 to February 2016) were systematically searched for publications on TKA with and without drainage. The following combinations of search terms were used to maximise search specificity and sensitivity: (total knee arthroplasty OR total knee replacement OR TKA OR TKR) AND (drainage OR drain). We also reviewed the reference lists of the retrieved articles to search for additional studies of interest that potentially met the study criteria despite not being captured in the electronic search.

### Eligibility criteria

A study was included if it met the following inclusion criteria: (1) contained results from randomised controlled trials (RCTs) (Level I evidence); (2) all patients underwent a primary, selective TKA; (3) compared closed drainage with nondrainage after TKA with regard to postoperative functional outcomes and/or complications; (4) no incorporation of perioperative auto-transfusion, reinfusion and vacuum aspiration systems; (5) written in English. Review articles, case reports, meeting abstracts, comments, letters, technical articles and expert opinions, along with animal and cadaver studies were excluded.

After excluding duplicates, two investigators independently screened the titles and abstracts to exclude irrelevant studies and identify relevant articles for full-text review. The two reviewers then independently reviewed the full text of the remaining articles and evaluated them against the inclusion/exclusion criteria to select articles for final inclusion. Disagreements regarding whether an article should be included or excluded were resolved by discussion, with arbitration by a third author if discrepancies remained.

### Risk of bias assessment

Two investigators independently assessed each included study using the Cochrane Collaboration tool for risk of bias, including random sequence generation, allocation concealment, blinding, incomplete outcome data, selective outcome reporting and other issues [15]. If all of the criteria were met, the study was considered to have a low risk of bias; if one or more of the criteria were partly met, the study was deemed to have an unclear risk of bias; if one or more of the criteria were not met, then the study was considered to have a high risk of bias. A risk-of-bias table was completed for each eligible study. Any differences were resolved by discussion, with arbitration by a third author if differences remained.

### Data extraction and statistics

Two reviewers independently extracted data from each included study. The data pertained to general information, surgical information, infection (any, superficial and/or deep infection), blood loss, blood transfusion (patient number and mean volume) and other outcomes (such as prolonged oozing of the wound, soft tissue ecchymosis, haematoma, deep venous thrombosis, postoperative VAS pain score and ROM). In cases of missing data, we attempted to contact the study authors to request it. Disagreements were resolved by discussion, with arbitration by a third author if disagreements remained. If trials could be pooled together for further analyses, statistical heterogeneity was assessed using the *Chi*^*2*^ test and *I*^*2*^ statistic to determine appropriateness for meta-analysis. The *Chi*^*2*^ < 0.10 or the *I*^*2*^ > 50 % was indicative of statistical heterogeneity. We conducted the meta-analysis using the Review Manager 5.2 software from the Cochrane Collaboration. The weighted risk ratios (RR) and 95 % confidence intervals (95 % CI) were calculated for the dichotomous variables while the weighted mean differences and accompanying 95 % CIs were calculated for the continuous variables. When there was no statistical evidence of heterogeneity, we adopted a fixed-effect model; otherwise, a random-effect model was chosen.

## Results

### Study characteristics

**Fig. 1:**
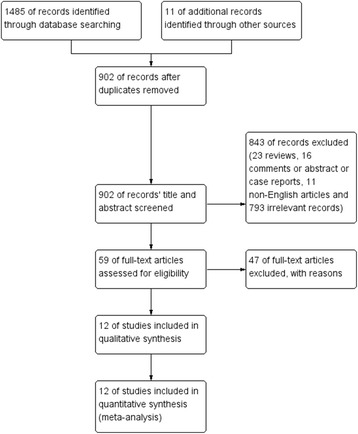
Flow diagram depicting the identification of the included studies

A flow diagram depicting the study identification is shown in Fig. 1. We identified 1496 potential articles (524 from PubMed; 206 from Embase; 250 from CENTRAL; 505 from Web of Science; and 11 from the reference lists). Of these, 12 articles totalling 889 TKAs met inclusion criteria for final review [2–11, 16, 17]. Tables 1 and 2 contain the summary general and surgical information on the included studies, respectively.

**Table 1:** General information of the selected 12 RCTs

References	TKAs (n)		Gender (male:female)		Mean age (years)		Diagnosis	Mean F/U		Results
	ND	D	ND	D	ND	D		ND	D	
Adalberth 1998 [17]	24	25	11:13	9:16	72	70	GA	4 m	4 m	−
Cao 2011 [6]	50	50	14:36	12:40	64.9	61.9	OA, RA	1y	1y	+
Crevoisier 1998 [10]	16	16	UC	UC	70	73	UC	UC	UC	−
Esler 2003 [11]	50	50	22:28	23:27	72.1	73.1	OA, RA	5y	5y	+
Fan 2013 [5]	40	40	16:24	16:24	66.5	66.5	OA	30d	30d	−
Holt 1997 [16]	68	69	20:48	24:45	69	70	UC	6w	6w	+
Jenny 2001 [9]	30	30	UC	UC	70	70	GA	UC	UC	−
Kim 1998 [3]	69	69	7:62	7:62	64	64	OA, RA	16 m	16 m	+
Liu 2014 [7]	6	12	1:5	3:9	67.2	67.2	OA	4w	4w	+
Niskanen 2000 [8]	19	20	5:14	4:16	71	70	OA	2 m	2 m	−
Omonbude 2010 [2]	38	40	23:15	20:20	68.4	71.1	OA	6w	6w	+
Ovadia 1997 [4]	26	32	6:20	7:25	69.7	73.7	OA, RA, AN	UC	UC	+

*Abbreviations: ND* nondrainage group, *D* closed drainage group, *F/U* follow-up, *UC* unclear, *GA* gonarthrosis, *OA* osteoarthritis, *RA* rheumatoid arthritis, *AN* avascular necrosis, *d* day, *w* week, *m* month, *y* year, + differences were found between the groups, − no difference was found between the groups

**Table 2:** Surgical and postoperative information of the selected 12 RCTs

References	Tourniquet	CD	DRT	Drain clamping	CPM	Thromboprophylaxis	VP	Transfusion standard
Adalberth 1998 [17]	Yes	Yes	24 h	UC	Yes	Enoxaparin	UC	Hb <90 g/L
Cao 2011 [6]	Yes	Yes	UC	UC	UC	LMWH	UC	Hb <10 g/dL
Crevoisier 1998 [10]	Yes	UC	48 h	UC	Yes	LMWH	UC	UC
Esler 2003 [11]	Yes	Yes	48 h	UC	UC	Aspirin	UC	Hb <10 g/dL
Fan 2013 [5]	UC	UC	24–48 h	UC	UC	LMWH	UC	UC
Holt 1997 [16]	Yes	Yes	48 h	UC	No	No	UC	Hb <8 g/dL
Jenny 2001 [9]	Yes	Yes	48 h	UC	UC	LMWH	UC	Hct <30 %
Kim 1998 [3]	Yes	UC	24 h	UC	UC	No	UC	UC
Liu 2014 [7]	Yes	Yes	24 h	Yes/4 h	Yes	Nadroparin	Yes	Hb <90 g/L
Niskanen 2000 [8]	Yes	Yes	NM	UC	UC	LMWH	UC	UC
Omonbude 2010 [2]	Yes	Yes	20.1 h	UC	UC	No	Yes	UC
Ovadia 1997 [4]	Yes	Yes	48 h	UC	Yes	Heparin	UC	Hb <8 g %

*Abbreviations: CD* compressive dressings, *DRT* drainage remove time, *CPM* continuous passive motion, *VP* venous pump, *UC* unclear, *LMWH* low molecular weight heparin, *Hb* haemoglobin, *Hct* haematocrit

### Risk of bias

**Fig. 2:**
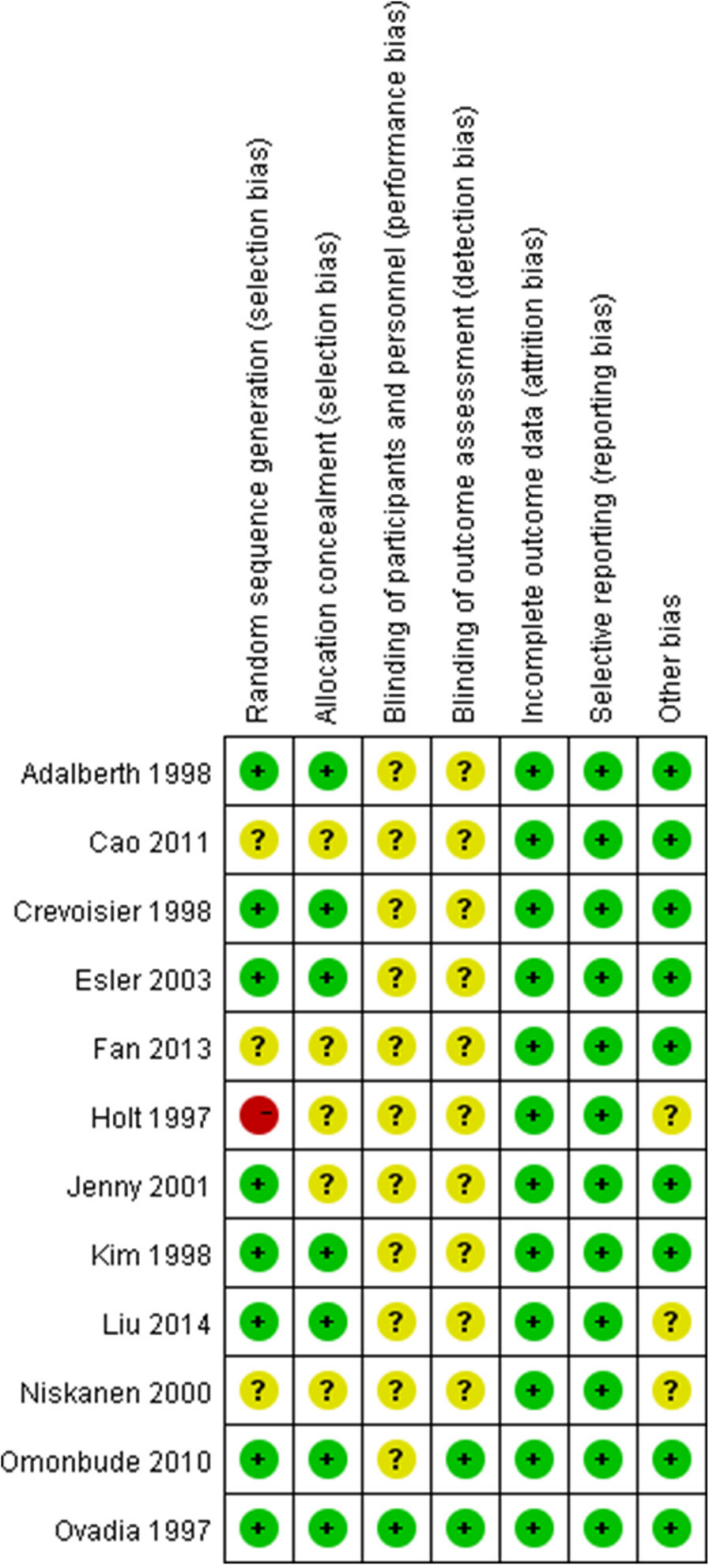
Risk of bias summary: review authors’ judgements about each risk-of-bias item for each included study

**Fig. 3:**
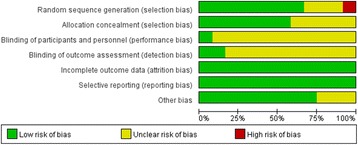
Risk of bias graph: review authors’ judgements about each risk of bias item presented as percentages across all included studies

The results of the quality assessment are shown in Figs. 2 and 3. Eight studies adequately described the correct randomisation, seven studies demonstrated sufficient allocation concealment, two studies described the blinding of outcome assessment and one study described the blinding of participants and personnel. All studies retained complete outcome data and avoided selective reporting, and nine studies appeared to be free of other potential sources of bias. As a result, the overall quality of the included studies was considered adequate, with the exception of only one study that demonstrated a high risk of bias (Fig. 2).

### Results of the meta-analysis

**Fig. 4:**
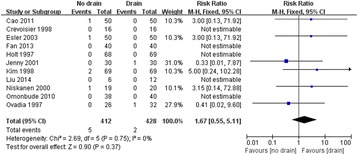
Forest plot of comparison: presence of infection (M-H: Mantel-Haenszel; CI: Confidence Interval; df: Degrees of freedom)

Table 3 shows the results of the meta-analysis. The primary outcome variable considered in this study was the presence of infection. The presence of infection after TKA was reported in eleven studies that included 840 TKAs. The heterogeneity among these studies was low, with a *Chi*^*2*^ of 0.75 and an *I*^*2*^ of 0 %. So a fixed-effect model was used for the meta-analysis. The nondrainage group had a higher rate of infection than the closed drainage group, with an RR of 1.67 (95 % CI, 0.55 to 5.11). However, the difference was not statistically significant (Fig. 4). Meanwhile, we also found no significant between-group differences in the rates of superficial and deep infections (Table 3).

**Table 3:** The results of the meta-analysis

Outcomes	Study (n)	TKA (n)	Heterogeneity		Effect model	RR / MD (95 % CI)
			*Chi* ^*2*^ (*p*)	*I* ^*2*^ (%)		
Infections						
Any infection	11	840	0.75	0	Fixed	1.67 [0.55, 5.11]
Superficial infection	4	275	0.59	0	Fixed	1.53 [0.30, 7.86]
Deep infection	3	256	0.82	0	Fixed	4.00 [0.45, 35.28]
Blood loss						
Total blood loss	3	178	<0.01	90	Random	−162.93 [−407.58, 81.72]
Hidden blood loss	2	118	<0.01	93	Random	−46.73 [−286.55, 193.08]
Number of transfusion	6	385	0.14	43	Fixed	0.53 [0.40, 0.69]
Volume of transfusion	2	149	0.34	0	Fixed	−180.30 [−268.32, −92.28]
Wound prolonged oozing	5	472	0.20	35	Fixed	2.31 [1.14, 4.68]
Soft tissue ecchymosis	5	432	<0.01	82	Random	2.23 [1.02, 4.88]
Operative time	2	118	0.30	8	Fixed	1.71 [−6.83, 10.25]
Wound haematoma	2	132	0.82	0	Fixed	0.45 [0.11, 1.84]
Deep venous thrombosis	6	474	0.31	5	Fixed	0.67 [0.25, 1.83]
VAS score						
postoperative day 1	2	98	0.34	0	Fixed	0.01 [−0.40, 0.41]
postoperative day 7	2	140	0.89	0	Fixed	0.16 [−0.55, 0.87]
postoperative day 14	2	140	0.47	0	Fixed	−0.44 [−0.56, 0.48]
Postoperative ROM						
postoperative day 2	2	78	0.17	47	Fixed	−2.60 [−6.77, 1.58]
postoperative day 3	2	118	<0.01	89	Random	−4.15 [−18.36, 10.06]
postoperative day 7	3	240	0.29	19	Fixed	−0.83 [−3.93, 2.27]
postoperative day 14	3	240	0.49	0	Fixed	−1.88 [−0.56, 1.29]
postoperative year 1	2	180	0.92	0	Fixed	0.21 [−1.57, 1.99]

*Abbreviations: RR* risk ratios, *MD* mean difference, *CI* confidence interval

**Fig. 5:**
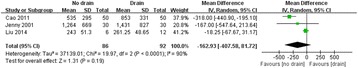
Forest plot of comparison: total blood loss (IV: Inverse Variance; CI: Confidence Interval; df: Degrees of freedom)

Four studies presented results for total blood loss [6, 7, 9, 11], of which three studies totalling 178 TKAs reported the total blood loss with a mean value (MV) ± standard deviation (SD) and were included in the meta-analysis [6, 7, 9]. After obtaining the heterogeneity among these studies (*Chi*^*2*^, < 0.01 and *I*^*2*^, 90 %), we chose to use a random-effect model. Analyses revealed greater total blood loss in the closed drainage group than in the nondrainage group, with a mean difference of −162.63 [95 % CI, −407.58 to 81.72], but the difference was not statistically significant (Fig. 5). Hidden blood loss, calculated by subtracting the amount of visible blood from total blood loss, was reported in two studies totalling 118 TKAs, and no significant difference was found between the two groups (Table 3). Only one study reported the intraoperative blood loss, and no significant difference was found between the two groups as well [7].

**Fig. 6:**
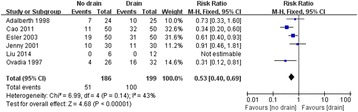
Forest plot of comparison: number of patients requiring a blood transfusion (M-H: Mantel-Haenszel; CI: Confidence Interval; df: Degrees of freedom)

In addition, the number of patients requiring blood transfusion was provided in six studies totalling 385 TKAs. It was noted that 51 (27.42 %) of 186 knees without drainage and 100 (50.25 %) of 199 knees with closed drainage required blood transfusion. No heterogeneity among these studies was found (*Chi*^*2*^, 0.14 and *I*^*2*^, 43 %); thus, fixed-effect model was used. The meta-analysis showed that the number of patient blood transfusions was lower in the nondrainage group than in the closed drainage group. This time the difference was statistically significant, with an RR of 0.53 (95 % CI, 0.40 to 0.69) (Fig. 6). The mean volume of blood transfusion was reported in seven studies, of which only two studies totalling 149 TKAs reported the data as MV ± SD. These data were then pooled together for meta-analysis [6, 17]. No heterogeneity among these studies was found (*Chi*^*2*^, 0.34 and *I*^*2*^, 0 %), and a fixed-effect model was used. Analyses showed that the mean volume of blood transfusion was significantly lower in the nondrainage than in the closed drainage group, with a mean difference of −180.30 (95 % CI, −268.32 to −92.28).

Prolonged oozing of the wound was reported in five studies totalling 472 TKAs. Overall, 20 of 232 (8.62 %) TKAs without drainage and 9 of 240 (3.75 %) TKAs with closed drainage presented prolonged oozing of the wound after surgery. The nondrainage group had a significantly higher rate of prolonged oozing of the wound, with an RR of 2.31 (95 % CI, 1.14 to 4.68). The meta-analysis also showed that soft tissue ecchymosis occurred significantly more often in the nondrainage group (RR, 2.23 and 95 % CI, 1.02 to 4.88). Additionally, there was no statistically significant difference in operative time, wound haematoma, deep venous thrombosis, postoperative VAS pain score or ROM between the two groups (Table 3).

## Discussion

The routine use of closed drainage for TKA has been suggested for many decades [1], but increasing clinical data has failed to show any advantage of postoperative closed drainage [18–20]. Many studies have compared closed drainage with nondrainage TKAs, but most of their samples were too small to allow for definite conclusions, particularly for outcomes with a low prevalence, such as infection [5, 7, 10]. Therefore, these data must be pooled together to determine differences between the two practices. The most important result from our study was finding no differences in the infection (total, superficial or deep infection) and blood loss (total, hidden or intraoperative blood loss) between the closed drainage and nondrainage groups, as well as in wound haematoma, deep venous thrombosis, postoperative VAS score and ROM.

Fear of infection is the primary reason for the use of closed drainage after TKA [21]. However, closed drainage has been associated with a higher risk of complications, such as retrograde infection [22]. Among the RCTs included in this study, four studies reported a higher rate [3, 6, 8, 11] while two studies reported a lower rate of infection in the nondrainage group relative to the closed drainage group [4, 9], but these differences were not statistically significant. Furthermore, the meta-analysis revealed no significant differences in any infection presence between the two groups. Subgroup analyses additionally showed no significant between-group differences in the rates of superficial and deep infections. Therefore, using closed drainage did not significantly reduce or increase the occurrence of infection after primary TKA.

It is well-known that most TKAs are conducted with the use of a tourniquet, and a little intraoperative blood loss is inevitable. So, the blood loss is mainly due to a large postoperative accumulation of blood within the joint space and muscle compartment. When the joint gap and muscle compartment fill with blood, bleeding ultimately stops because of the tamponade effect [23]. If a drain is then placed, theoretically, the tamponade effect will weaken or disappear, causing further bleeding into the drain until it is removed. However, this meta-analysis found no significant between-group differences in total or hidden blood loss, although the total blood loss was more, and the hidden blood loss was less, in the closed drainage group. Clinically, the postoperative bleeding is thought to occur during the early stage after surgery, and as a result, many studies have theorised that postoperative bleeding might be decreased by the temporary clamping of drains postoperatively [24, 25], the use of a tourniquet intraoperatively [26, 27], and/or intravenous and/or local use of tranexamic acid perioperatively [28], which is discussed below.

Although we found no differences in total and hidden blood loss between closed drainage and nondrainage TKAs as previously discussed, this meta-analysis suggests that both the patient number and mean volume of blood transfusions after TKA were statistically lower in the nondrainage group. Data from two studies totalling 149 TKAs were pooled together for the analysis of transfusion volume in this study. Analyses ultimately revealed greater total blood loss in the closed drainage group. Although the difference was not statistically significant, considerable differences between each patient’s blood loss and poorly controlled transfusion standards in the closed drainage group may be the reasons. Among the included RCTs, two studies reported a haemoglobin decrease, and no between-group difference was found [11, 16]. One of those studies, however, reported that the total blood loss and the blood transfusion rate were significantly higher in the closed drainage group [11]. When taking into account the rare risks and complications following blood transfusion, such as infection, haemolytic transfusion reactions, transfusion-related lung injury and health care costs for patients [12, 29–31], the reduction of blood transfusions is of value. However, in our department, where approximately 1500 TKAs were performed in 2015, closed drainage was routinely used before 2015, and we found that almost no patients needed a blood transfusion postoperatively, especially in the past five years. Taken together with the negative results for infection and blood loss, the higher rate of blood transfusion revealed in this meta-analysis might be insufficient to reject the use of closed drainage after primary TKA. Furthermore, a randomised controlled trial with a large sample is currently being conducted in our department, and we hope it will provide more evidence on this topic.

It is thought that the collection of blood and the formation of haematomas in the knee after TKA could impair wound healing, increase the risk of deep infection, and cause pain and stiffness with resultant delays in rehabilitation and extended hospital stays. Closed drainage theoretically reduces the postoperative collection of blood in a closed space and prevents haematoma formation [11], and surgeons are always meticulous in exploring better methods of closed drainage, such as the temporary clamping of drainage, which may create a tamponade effect and control postoperative blood loss. A recent meta-analysis revealed that non-continuous drainage can result in less haemoglobin loss (especially with four- to six-hours of drain clamping) and less visible postoperative blood loss with no increased risk of postoperative complications compared with continuous drainage [24]. Furthermore, Yildiz et al. reported that drain clamping (6 h) combined with late tourniquet release (after skin closure) reduced postoperative blood loss in TKR surgery [32]. Similarly, Chareancholvanich et al. found that drain clamping (3 h) combined with tranexamic acid administration could reduce postoperative blood loss and blood transfusion after TKA [25]. Thus, improving the use of closed drainage in primary TKA, such as temporary clamping combined with late tourniquet release or tranexamic acid as discussed above, might provide better results.

The reluctant advantages of the use of closed drainage demonstrated by this meta-analysis are the logical and effective ways closed drainage can reduce prolonged wound oozing and soft tissue ecchymosis after primary TKA. However, there were no differences noted in the infection rate and blood loss between the two practices, and careful wound care might be helpful in improving the prolonged oozing of the wound in nondrainage patients. Therefore, it seems that there are no clear benefits and drawbacks to the use of closed drainage after primary TKA.

One meta-analysis within the past three years, published in English, evaluated the efficacy and safety of closed drainage [33]. Only six RCTs published before 2010 were included, and three outcomes, postoperative haemoglobin drop, ROM and knee circumference, were analysed. The only significant finding of that meta-analysis was that the haemoglobin drop was significantly higher in the closed drainage group than the nondrainage group. In this review, we tried to capture all RCTs published in English to date, which, at present, provide the best source of information on this topic. As a result, we have included six additional RCTs and analysed more outcomes. However, this study also has some limitations. First, one of the most important biases to minimise for determining the quality of the evidence, the blinding, was not accomplished in most of the reviewed studies. Secondly, other confounding factors, such as the use of a tourniquet and the temporary clamping of the drainage, as well as the removal time of the drainage and the standard for postoperative blood transfusion within the included studies, might have also affected the results. Despite these limitations, we were able to include and analyse 12 RCTs that were published within the past 18 years. The overall quality of these studies was adequate, suggesting that these studies are comparable and that pooling them is advisable.

## Conclusions

No significant difference in infection rate or blood loss was found between the closed drainage and nondrainage TKAs, there appears to be no clear benefit or drawback to the use of closed drainage after primary TKA. Improving the use of closed drainage, such as temporary clamping, or combining it with late tourniquet release or tranexamic acid, might provide better results.

### Availability of data and materials

All data and materials are contained within the manuscript.

## Abbreviations

CI: confidence interval
MV: mean value
RCT: randomized controlled trials
ROM: range of motion
RR: risk ratio
SD: standard deviation
TKA: total knee arthroplasty
TKR: total knee replacement
VAS: visual analogue scale
